# B1486 is a highly potent VprBP inhibitor capable of blocking osteoclast differentiation and bone resorption

**DOI:** 10.1016/j.jbc.2026.113524

**Published:** 2026-09-08

**Authors:** Sungmin Kim, Yonghwan Shin, Nikhil B. Ghate, Woojin An

**Affiliations:** Department of Cancer Biology, Norris Comprehensive Cancer Center, University of Southern California, Los Angeles, California, USA

**Keywords:** protein kinase, phosphorylation, histone, inhibitor, osteoclast

## Abstract

Bone is a dynamic tissue that is continuously remodeled by tightly coordinated actions of bone-resorbing osteoclasts and bone-forming osteoblasts. Uncontrolled osteoclastogenesis has a severe impact on skeletal health and thus represents an important therapeutic target in osteolytic diseases. VprBP is a recently identified kinase and plays an important role in stimulating osteoclast differentiation and bone resorption through histone H2AT120 phosphorylation (H2AT120p). We previously developed B32B3 as the first VprBP inhibitor, but its high IC_50_ value has been a major challenge in using it for osteoclast-triggered bone diseases. In this study, we designed and characterized a series of small molecule compounds derived from B32B3 to identify a novel second-generation inhibitor of VprBP. Our initial screening of B32B3 derivatives identified three compounds, designated as B1486, B3703, and B6756, that are effective in interfering with VprBP kinase activity toward H2AT120p and RANKL-induced differentiation of osteoclast precursor cells (OCPs). However, when their cellular efficacy was systematically accessed over different concentration ranges, B1486 was more potent than B3703 and B6756 at hindering VprBP function in the process of OCP cell differentiation. Consistent with these *in vitro* findings, B1486 showed significantly improved inhibitory activity against VprBP-driven bone resorption and low bone mass phenotypes in our preclinical studies using zebrafish and mouse models. Taken together, our results indicate that B1486 is a highly potent inhibitor for blocking VprBP-mediated H2AT120p and osteoclastogenic gene silencing, as well as for overcoming osteoporosis generated by excessive osteoclast differentiation.

VprBP, also called DCAF1, was originally identified as a protein that can interact with the HIV-1 accessory protein viral protein R (Vpr) and hence was named Vpr-binding protein (VprBP) (1, 2). Since then, VprBP has been mainly characterized as a substrate receptor acting in the DDB1-Cul4-ROC1 E3 ubiquitin ligase complex and controlling cell growth and differentiation (3, 4, 5, 6, 7, 8, 9, 10). However, we unexpectedly found that VprBP has intrinsic kinase activity and phosphorylate threonine 120 of histone H2A (H2AT120) (11). H2AT120 phosphorylation (H2AT120p) is necessary for VprBP-driven gene silencing, because VprBP kinase-dead mutation (VprBPK194R) and H2AT120p-blocking mutation (H2AT120A) eliminate the ability of VprBP to establish an inactive chromatin state in our functional assays using cancer cells (11, 12, 13, 14). Adding support to this observation, our genome-wide transcriptomic analyses uncovered gene-selective corepressor function of VprBP specifically targeting growth regulatory genes in several cancer types (11, 12, 13). A pro-oncogenic role for VprBP is further supported by the observation that VprBP also phosphorylates EZH2 at T367 (EZH2T367p) in colon cancer cells (15) and p53 at S367(p53S367p) in DNA damage-response cascade (16). All these studies established VprBP function in oncogenic gene silencing but left open the question of whether VprBP plays a similar role in other cellular processes. In this regard and of special relevance to the current study, our continued investigation revealed a key role played by VprBP in RANKL-induced differentiation of osteoclast precursor (OCP) cells to mature osteoclasts with bone-resorbing activity (17). Under RANKL treatment conditions, VprBP is highly expressed and potentiates OCP cell differentiation in a manner dependent on its kinase activity toward H2AT120p (17). Moreover, our RNA-seq analysis indicated that targeting and silencing genes encoding negative regulators of osteoclastogenesis reflects the functional contribution of VprBP to RANKL-induced OCP cell differentiation (17).

In light of the tumorigenic role of VprBP identified in our initial study, we developed a cell-permeable, small-molecule inhibitor, named B32B3, capable of selectively targeting VprBP and blocking its kinase activity in cancer cells (11). Considering the functional significance of VprBP-mediated H2AT120p in osteoclastogenic gene silencing (17), one would also expect anti-osteoclastogenic effects of B32B3. We found this expectation to be indeed the case; B32B3 treatment generated almost complete loss of H2AT120p and attenuated osteoclast differentiation at levels similar to those observed with VprBP knockdown (17). This first-generation inhibitor retained high potency against VprBP and proved useful for impairing osteoclast formation in zebrafish and mouse models (17). In this preclinical study, B32B3 treatment also resulted in an apparent decrease in RANKL-induced bone resorption, thereby ameliorating bone destruction and defects. These findings suggest that B32B3 treatment may be effective in preventing bone diseases caused by VprBP-induced excessive bone resorption. However, the moderate cellular potency of B32B3 has been a central challenge in its use when inhibiting VprBP kinase activity is clinically useful. As such, improvements in the potency and efficacy of B32B3 are needed before VprBP inhibition approaches are considered therapeutically.

In the present study, we sought to develop second generation VprBP inhibitors that exibit improved activities and possess drug-like properties in OCP-induced cells. By screening a library of 211 B32B3-like compounds, we identified B1486 as retaining extremely high potency in blocking H2AT120p, reactivating anti-osteoclastogenic genes, and impairing osteoclast differentiation. More intriguingly, B1486 almost completely inhibited RANKL-induced bone defects and effectively ameliorated pathological bone resorption in pre-clinical models.

## Results

### B1486 inhibits VprBP-driven osteoclastogenesis and H2AT120p

To identify a novel B32B3 mimicking small molecule inhibitor with higher potency, we used the Chemically Advanced Template Search (CATS) distance metric for the prediction of their potential activities (18, 19). From CATS-based designs of B32B3 analogues, 211 compounds containing B32B3-like scaffold were synthesized and screened for an ability to attenuate the differentiation capacity of OCP cells into mature osteoclasts in response to RANKL treatment for 5 days. In the initial phase of analogue screen, five analogues were randomly pooled together at 100 nM each and tested in OCP-induced cell culture at the total concentration of 500 nM. The compound pools 6, 11, and 13 completely abrogated the differentiation potential of OCP-induced cells (Fig. 1*A*), while other pools showed no or only modest effects on OCP cell differentiation (not shown), as monitored by TRAP staining. In these cellular assays, H2AT120p was totally abolished following treatments with the compound pools 6, 11, and 13 without altering VprBP expression levels (Fig. 1*B*). In order to identify the most potent compound in the three active pools, 15 compounds of the three pools were individually retested in OCP-induced cell culture at the final concentration of 100 nM. As shown in Figure 1*C*, RANKL-induced osteoclast differentiation was fully inhibited by B1486 from Mix 6, B6756 from Mix 11, and B3703 from Mix (Figs. 1*C* and S1, *A* and *B*). In contrast, OCP-induced cells were relatively resistant when being treated with each of 12 other compounds at 100 nM (Figs. 1*C* and S1, *A* and *B*).

**Figure 1:**
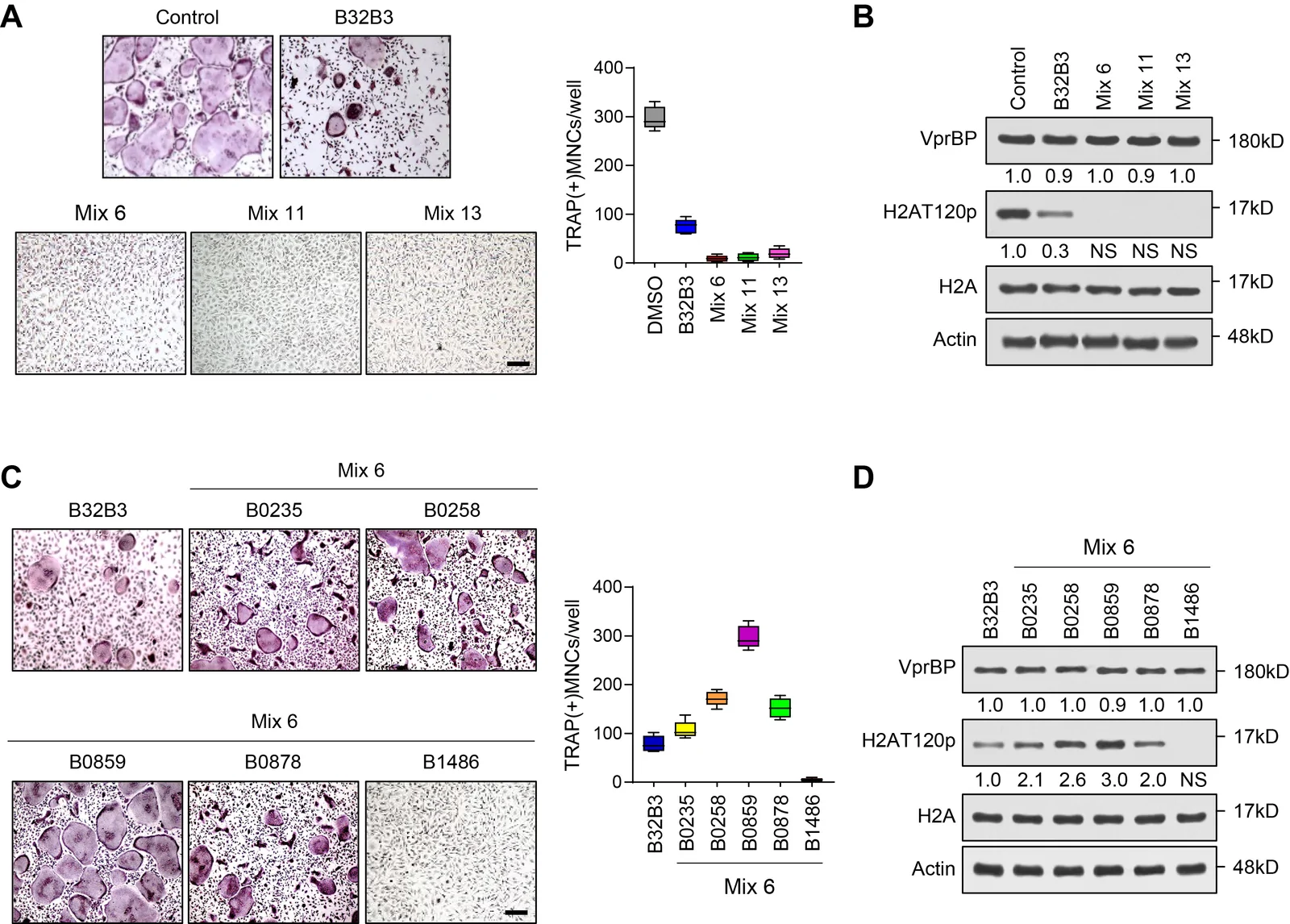
**B1486 is very effective in impairing osteoclast differentiation.***A*, pooled compound plates were prepared by randomly combining five analogues at 100 nM each (total 500 nM per well) in 24-well plates containing 30 ng/ml macrophage colony-stimulating factor (M-CSF) and 50 ng/ml receptor activator of nuclear factor kappaB ligand (RANKL). Osteoclast precursor (OCP) cells were seeded on these plates and cultured for 5 days as previously described (17). Cells were then fixed with formaldehyde, stained for TRAP (tartrate-resistant acid phosphatase), and photographed under a light microscope (*left panel*; scale bar, 100 μm). Shown are the representative results of three independent biological experiments using compound mixes 6, 11, and 13, each consisting of at least n = 5 technical replicates. TRAP-positive cells containing three or more nuclei were counted as osteoclasts (*right panel*). The band intensities of VprBP and H2AT120p were quantified using Image J software (v1.53k) after normalization to Actin and H2A signals. The corresponding optical densities are shown below. NS indicates no signal. Data represent the mean ± SD of independent biological triplicate experiments. All experiments were performed under identical conditions, including the same cell batch, vehicle, and treatment schedule. *B*, OCP-induced cells were cultured as in (*A*), and whole cell lysate and chromatin fractions were prepared and analyzed by Western blotting with VprBP, H2AT120p, and H2A antibodies to assess VprBP-mediated H2AT120p. The band intensities of VprBP and H2AT120p were quantified using Image J software (v1.53k) after normalization to Actin and H2A signals. The corresponding optical densities are shown below. NS indicates no signal. Data are representative of three independent biological experiments, each consisting of at least five technical replicates. *C*, based on the initial screening in (*A*), OCP-induced cells were treated with 100 nM of each indicated mix 6 compound for 5 days, and TRAP-stained (*left panel*; scale bar, 100 μm) and counted (*right panel*). Representative images of osteoclasts are shown. All experiments were performed under identical conditions, including the same cell batch, vehicle, and treatment schedule. *D*, whole-cell lysate and chromatin fractions were prepared and analyzed by Western blotting as in (*B*). Data are representative of three independent biological experiments.

For further characterization of B1486, B6756, and B3703, we titrated them over the concentration range of 0 to 100 nM in OCP cell differentiation assays. The B1486 dose dependently generated abortive OCP cell differentiation with a full inhibitory activity at low nanomolar concentrations (Fig. 2*A*). Under the same treatment conditions, B6756 and B3703 were much less potent in attenuating OCP cell differentiation compared to B1486 without showing any low nanomolar inhibitory activities (Fig. S3, *A*–*C*). Consistent with these results, B1486 more potently inhibited VprBP-mediated H2AT120p than B6756 and B3703 in OCP-induced cells, as determined by Western blotting of nuclear fractions (Figs. 2*B* and S4, *A* and *B*).

**Figure 2:**
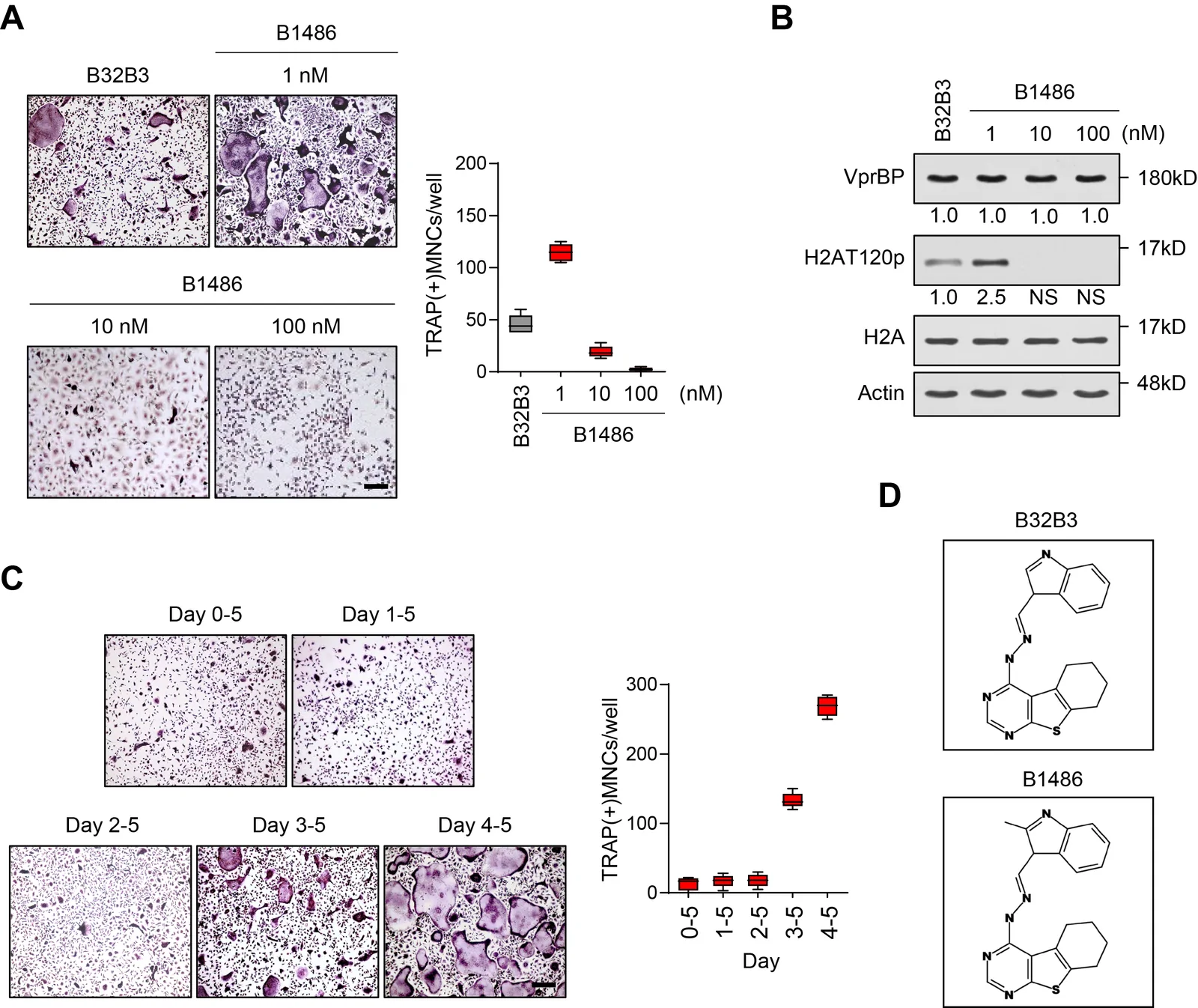
**B1486 inhibits osteoclastogenesis with low nanomolar potency.***A*, OCP cells were cultured with 30 ng/ml M-CSF and 50 ng/ml RANKL to induce differentiation in the presence of B1486 at indicated concentrations or 100 nM B32B3 for 5 days, The cells were then fixed with formaldehyde, TRAP-stained, and photographed under a light microscope (*left panel*; scale bar, 100 μm). Data are representative of three independent experiments. TRAP-positive cells containing three or more nuclei were counted as osteoclasts (*right panel*). Data represent the mean ± SD of three independent biological experiments, each consisting of at least five technical replicates. All experiments were performed under identical conditions, including the same cell batch, vehicle, and treatment schedule. *B*, OCP-induced cells were cultured for 5 days as in (*A*). Whole cell lysate and chromatin fractions were prepared and analyzed by Western blotting with VprBP, H2AT120p, and H2A antibodies. The band intensities of VprBP and H2AT120p were quantified using Image J software (v1.53k) after normalization to Actin and H2A signals. The corresponding optical densities are shown below. NS indicates no signal. Data are representative of three independent biological experiments. *C*, OCP-induced cells were treated with 10 nM B1486 as described in (*A*) but under five different exposure time windows: day 1 to 5, day 2 to 5, day 3 to 5, day 4 to 5 and day 5 only. TRAP-stained OCP cells were analyzed under a light microscope and counted as described in (*A*). Data are representative of three independent experiments, each consisting of at least five technical replicates. All experiments were performed under identical conditions, including the same cell batch, vehicle, and treatment schedule. *D*, molecular structures of B32B3 and B1486.

Also, our RT-qPCR analysis detected lower expression of NFATc1, a master regulator of osteoclast differentiation, after treating OCP-induced cells with B1486 (Fig. S5). Because VprBP levels and OCP cell viability were unaffected or minimally influenced after 5 days of B1486 treatment (Figs. 2*B* and S6, *A*–*C*), B1486 must exhibit suppressive effects on OCP cell differentiation, mainly targeting VprBP kinase activity. Supporting these observations, B1486 also showed a more than 10-fold higher potency compared to B6756 and B3703 in recombinant VprBP kinase assays using nucleosome substrates (Fig. S7, *A*–*C*). As sumerized in Fig. S8, more detailed dose–response kinase assays based on this initial observation identified the IC_50_ value of B1486 against VprBP to be 3.8 nM. Futhermore, in measuring the selectivity of B1486 with 15 other kinases, we found that B1486 specifically targeted and inhibited VprBP kinase activity (Fig. S9). Recent evidence from our work indicates that VprBP is expressed from day 3 of RANKL induction and contributes to the middle-to-late stages of osteoclast differentiation through H2AT120p. Such stage-specific function of VprBP-mediated H2AT120p would predict that B1486 treatment at the middle stage is still efficient in establishing the inactive state of osteoclastogenesis. Expectedly, when OCP cells were exposed to 10 nM B1486 0, 1, 2, 3, or 4 days after RANKL induction, B1486 treatment from days 1 and 2, but not from days 3 and 4, inhibited OCP cell differentiation in the level as that observed with B1486 treatment from day 0 (Fig. 2, *C* and *D*).

### B1486 reactivates VprBP target genes in OCP-induced cells

Knowing that B1486 is highly effective in abrogating osteoclastogenic function of VprBP, we next sought to determine the effects of B1486 on VprBP function at target genes. Toward this end, we exposed OCP-induced cells to B1486 and measured mRNA levels of 10 representative target genes encoding negative regulators of osteoclast differentiation. Congruent to our published data (17), RT-qPCR analysis demonstrated that the selected target genes are inactivated in OCP cells upon RANKL induction (Fig. 3*A*). However, when OCP-induced cells were treated with B1486 at a concentration of 10 nM, the active state of those anti-osteoclastogenic genes was established, leading to a significant increase in their transcription levels (Fig. 3*A*). As there was no change in transcription status of the control gene RPL23 in B1486-treated cells, VprBP-induced gene silencing was mainly targeted by B1486. This observation is also consistent with the view that B1486 inhibits differentiation potential of OCP-induced cells by establishing active states of VprBP target genes.

**Figure 3:**
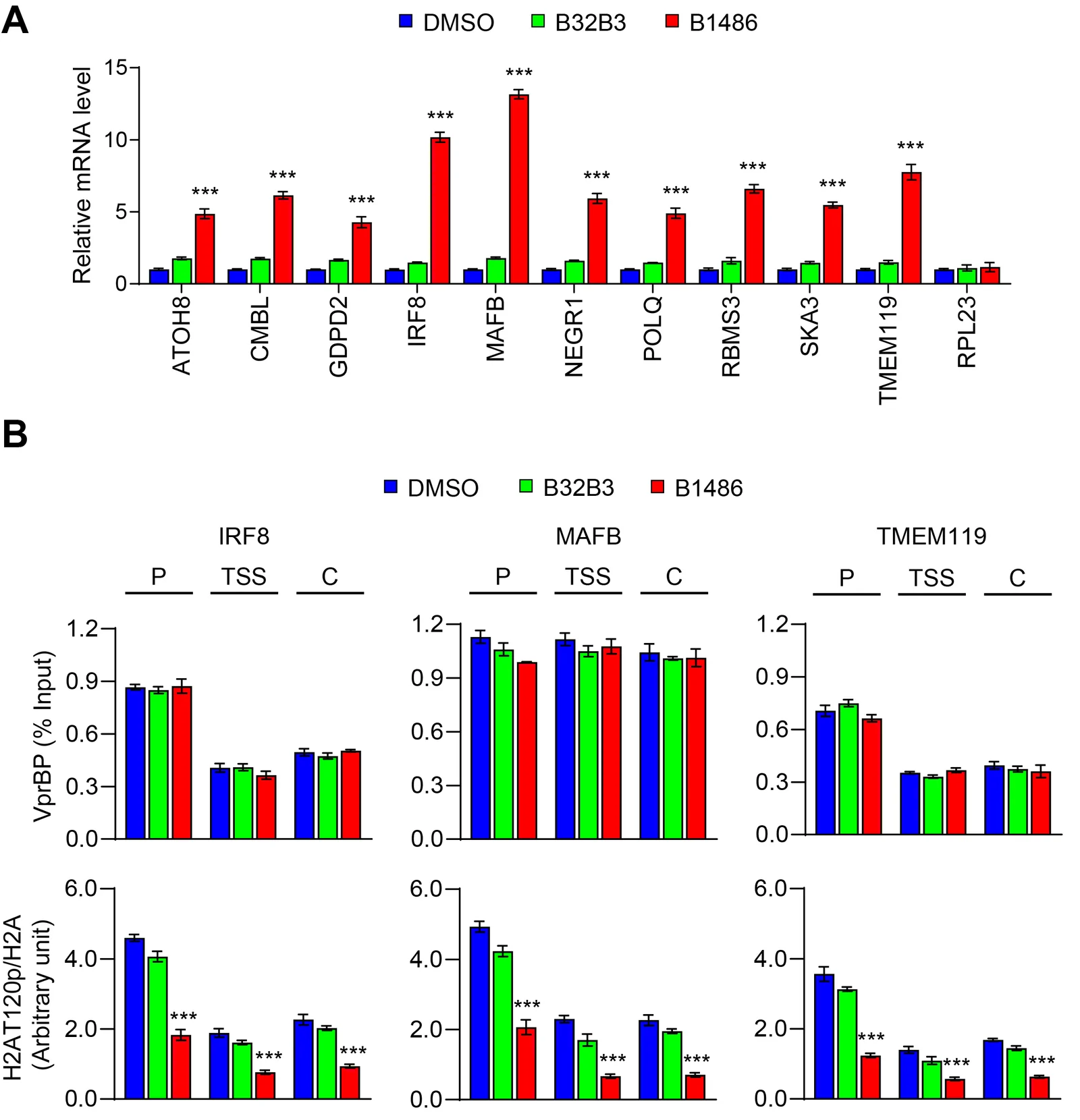
**B1486 antagonizes VprBP-mediated gene silencing in OCP-induced cells.***A*, OCP-induced cells were treated with VprBP inhibitors B32B3 and B1486 at final concentrations of 100 nM and 10 nM, respectively. RNA samples were prepared, and VprBP target gene expression was analyzed by RT-qPCR using primers listed in Table S1. All transcription levels were normalized to those of GAPDH. Data represents the mean ± SD of three independent biological experiments, each consisting of at least five technical replicates. *p* values were calculated using two-way ANOVA with *post hoc* Tukey’s test for multiple comparisons. ∗∗∗*p* < 0.001 *versus* B32B3. *B*, ChIP assays were performed on OCP-induced cells described in (*A*) with VprBP, H2AT120p, and H2A antibodies. The ChIP DNAs were analyzed by real-time PCR with primer pairs amplifying the promoters (P), transcription start site (TSS), and coding regions (C) of IRF8, MAFB, and TMEM119 genes. Primers used are listed in Table S2. Data were expressed as mean ± SD of three independent biological experiments, each consisting of at least five technical replicates. *p* values were calculated using two-way ANOVAwith *post hoc* Tukey’s test for multiple comparisons. ∗∗∗*p* < 0.001 *versus* control; ^###^*p* < 0.001 *versus* B32B3.

Although the abovementioned results indicate the selective potentiation of VprBP target genes upon treatment with B1486, we could not discriminate between direct and indirect effects of B1486 in this analysis. We therefore used chromatin immunoprecipitation (ChIP) assays to examine VprBP occupancy and H2AT120p levels at target genes. Among the 10 target genes we analyzed by RT-qPCR above, IRF8, MAFB, and TMEM119 were selected for our ChIP analysis, because they showed the most significant upregulation in response to B1486 treatment (Fig. 3*B*) and because they play critical roles in controlling osteoclast differentiation (17). Crosslinked chromatin was isolated from control and B1486-treated OCP-induced cells, and the precipitated DNA was amplified by quantitative PCR (qPCR) with three primer pairs targeting promoter (P), transcription start site (TSS), and coding region (CR) of the genes. We observed higher levels of VprBP and H2AT120p at the promoter region compared to the coding region and TSS, although the precipitation levels were slightly different for the three target genes (Fig. 3*B*). When ChIP experiments were performed in B1486-treated cells, the levels of VprBP at the target genes remained unchanged. However, almost complete loss of H2AT120p ChIP signals was detected at the target genes (Fig. 3*B*), results indicative of the capability of B1486 to prevent VprBP-mediated H2AT120p. Overall, these and the above-noted data suggest that B1486 inhibits RANKL-induced osteoclast differentiation by attenuating VprBP-mediated H2AT120p and re-activating anti-osteoclastogenic genes.

### B1486 blocks pathological bone resorption

To further confirm the *in vitro* observations mentioned previously, we next wanted to examine the *in vivo* pharmacological effects of B1486 on bone metabolism, especially osteoclastic bone resorption. Zebrafish is an excellent model organism for addressing this question due to its advantages such as large spawning capacity, rapid external development, and transparent body. If zebrafish larvae at 6 days post-fertilization (dpf) are treated with 25 μM prednisolone (PDS) for 3 days, we can artificially induce bone resorption and characterize possible effects of inhibitor treatment on bone stability by calcein staining. Our recent study successfully used this model system to show that B32B3 inhibits prednisolone (PDS)-induced osteoporosis at a final concentration of 2 μM (17). Given that B1486 displays about 50-fold higher potency than B32B3 in our cellular assessments, it was reasonable to expect an anti-resorptive efficacy of B1468 at much lower doses. Treating zebrafish larvae at 6 dpf with 25 μM PDS for additional 3 days accelerated bone resorption as evaluated by calcein staining. Notably, however, exposure to 40 nM B1486 significantly ameliorated the bone development defect arising from PDS treatment by impeding bone resorption (Fig. 4, *A*–*C*). Along with our demonstration of B32B3 being minimally effective at the same concentration, these results emphasize that B1486 is much more potent than B32B3 in interfering with PDS-induced bone resorption in zebrafish.

**Figure 4:**
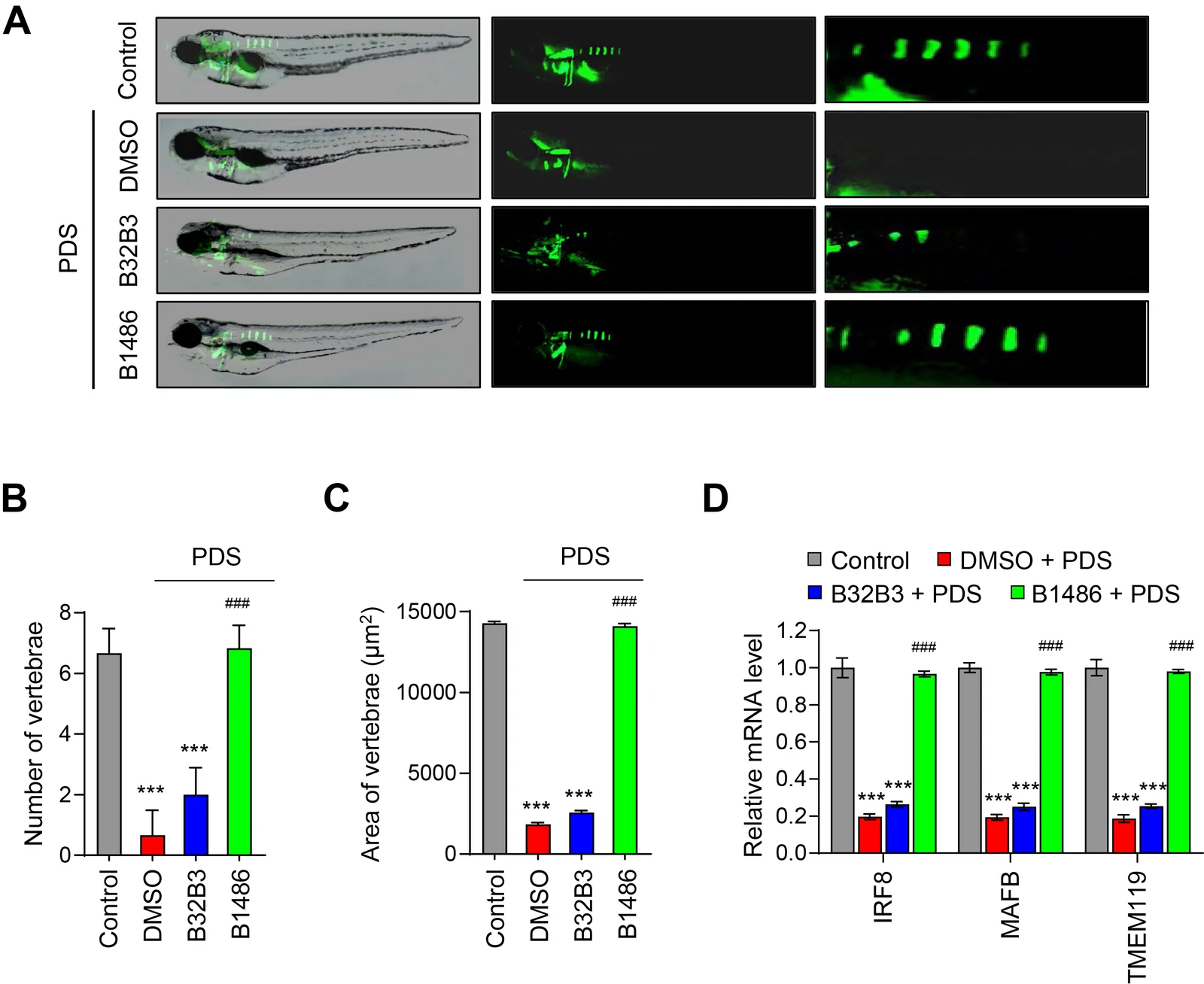
**B1486 attenuates bone resortion in zebrafish***. A*, Zebrafish larvae were treated with the indicated concentrations of B32B3 or B1486 in the presence or absence of 25 μM prednisolone from 6 days post-fertilization (dpf) to 9 dpf. Skeletal mineralization was evaluated by calcein staining. Data are representative of three independent biological experiments (n = 12 biologically independent larvae per experiemnt). *B*, zebrafish were treated as in (*A*), and the calcification degree was measured by counting the number of vertebral segments in zebrafish larvae. Data represent the mean ± SD of three independent biological experiments. (n = 12 biologically independent larvae per experiemnt). *p* values were calculated using one-way ANOVAwith *post hoc* Tukey’s test for multiple comparisons. ∗∗∗*p* < 0.001 *versus* control; ^###^*p* < 0.001 *versus* B32B3. *C*, zebrafish were treated as in (*A*), and the relative bone mineral density was calculated by measuring the areas of the first five stained vertebrae. Data represent the mean ± SD of three independent biological experiments. (n = 12 biologically independent larvae per experiemnt). *p* values were calculated using one-way ANOVAwith *post hoc* Tukey’s test for multiple comparisons. ∗∗∗*p* < 0.001 *versus* control; ^###^*p* < 0.001 *versus* B32B3. *D*, total RNA was extracted from PDS/B32B3 or B1486-treated 9 dpf zebrafish larvae, and the expression levels of IRF8, MAFB, and TMEM119 genes were quantified by RT-qPCR using primers listed in Table S1. Data represent the mean ± SD of three independent biological experiments. ∗∗∗*p* < 0.001 *versus* control; ^###^*p* < 0.001 *versus* B32B3 + PDS.

To further assess anti-resorptive efficacy of B1486 *in vivo*, B1486 was administered to the periosteal regions of calvarial bones in RANKL-treated mice every other day for 2 weeks. In good agreement with our recent reports (17), micro-computed tomography (micro-CT) analysis demonstrated that calvarial injection of RANKL elevates bone resorption and thus induces bone destruction in mouse calvaria (Fig. 5, *A* and *B*). Meanwhile, administration of B1486 to mice at a dose of 0.1 mg kg^–1^ stimulated bone repair and regenerative capacities at periphery and center of RANKL-induced bone defects (Fig. 5, *A* and *B*). Our analysis also detected a substantial increase in bone mineral density (BMD) and bone volume-to-tissue volume ratio (BV/TV) in mice treated with B1486 under RANKL-induced condition compared to RANKL alone-treated mice (Fig. 5*C*). The observed changes in BMD and BV/TV after B1486 treatment were due to reduced osteoclast differentiation, because lower numbers of TRAP-positive osteoclasts were detected in B1486-treated mice as compared to control and B32B3-treated mice in our histomorphometric analysis (Fig. 5*E*). Moreover and as shown in Figure 5*D*, the fact that mice treated with B1486 display smaller cavities in bone strongly argues that B1486 inhibits osteoclast-mediated bone resorption, thereby enhancing bone stability. Mirroring the results from zebrafish, we failed to observe any significant changes in RANKL-induced calvarial bone defect in mice treated with B32B3 at a dose of 0.1 mg kg^−1^ which is ∼50 times lower than the effective dose we identified in recent studies (Fig. 5, *A*–*C*). Considering the importance of H2AT120p in VprBP-driven transcriptional silencing of anti-osteoclastogenic genes, we also examined whether B1486-induced blocking of VprBP kinase activity also affects target gene expression in zebrafish and mice. As summarized in Figures 4*D* and 5*D*, B1486 treatment generated, albeit to a somewhat varying extent, an active state of VprBP target genes in those two *in vivo* models. As a validation of its anti-osteoclastogenic effects, treating zebrafish and mice with B1486 also reduced the expression of NFATc1 osteoclast marker gene (Fig. S10, *A* and *B*). These results thus reinforce our conclusion that B1486 has negative impact on bone resorption and stimulates skeletogenesis by blocking VprBP-mediated H2AT120p as well as reactivating anti-osteoclastogenic genes.

**Figure 5:**
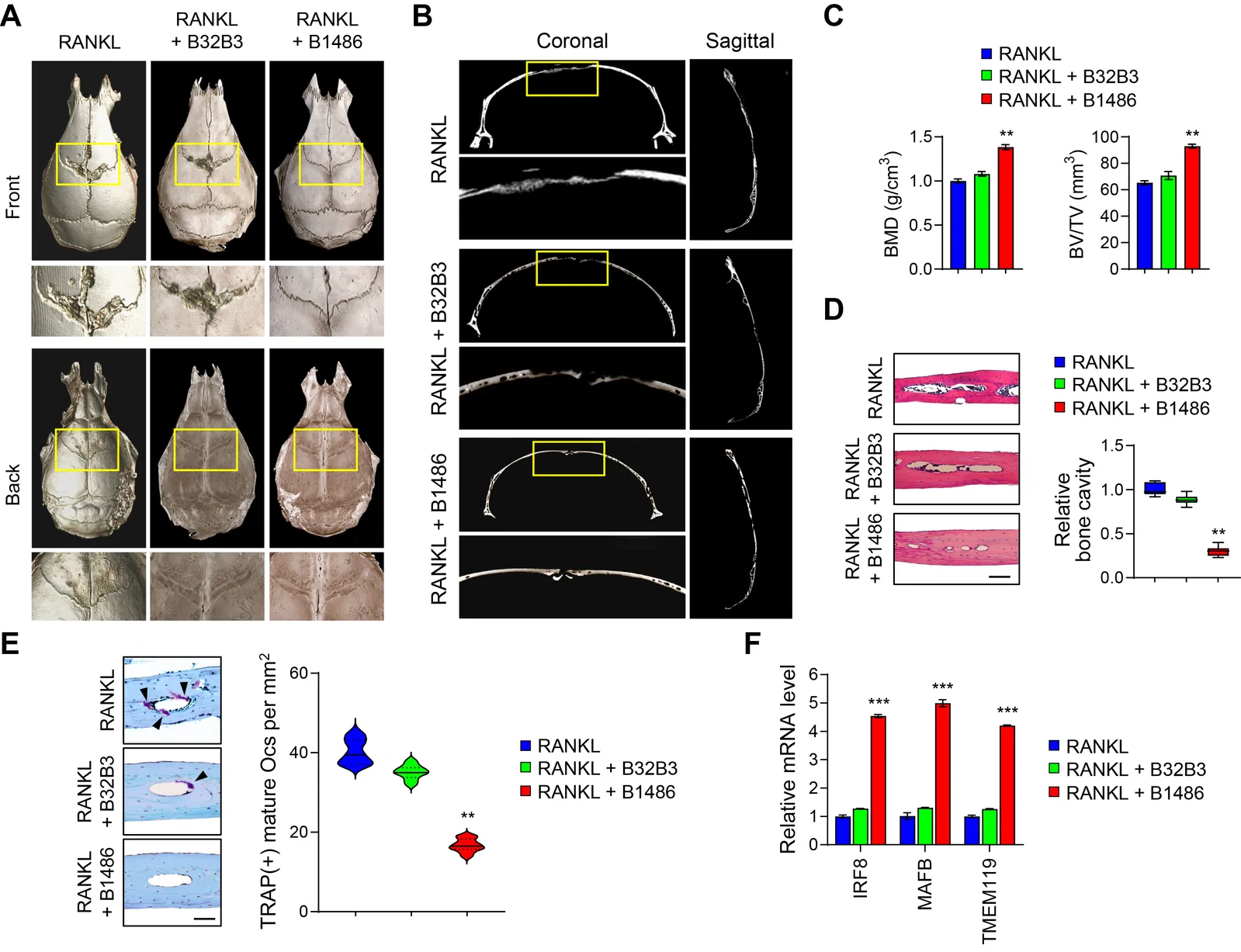
**B1486 interferes with bone destruction in mice.***A*, male C57BL/6 mice (8 weeks old) were treated with RANKL alone (RANKL) or RANKL together with B32B3 (0.1 mg kg^–1^; RANKL + B32B3) or B1486 (0.1 mg kg^–1^; RANKL + B1486) every other day for 14 days, receiving RANKL alone on the intervening days. At the end of 14 days treatments, mice were sacrificed, and calvarial bones were subjected to micro-computed tomography (micro-CT) analysis. Three-dimensional micro-CT images representing frontal and posterior views including magnified coronal sutures were acquired and are presented as representative illustrations in this study. n = 6 biologically independent mice per experiment. *B*, 2D coronal and sagittal slice images, including magnified views of the coronal sutures, were generated from the micro-CT images shown in (*A*). Representative images are shown (n = 6 biologically independent mice per expreiment). *C*, bone mineral density (BMD) (*left panel*) and bone volume (BV) (*right panel*) per tissue volume (TV) were quantified from micro-CT images. Data are shown as mean ± SD (n = 6). *p* values were calculated using one-way ANOVAwith *post hoc* Tukey’s test for multiple comparisons. ∗∗∗*p* < 0.001 *versus* RANKL + B32B3. *D*, calvarial bones from C57BL/6 mice treated with RANKL alone (RANKL), or RANKL together with B32B3 (0.1 mg kg^–1^; RANKL + B32B3) or B1486 (0.1 mg kg^–1^; RANKL + B1486) were fixed, embedded, and subjected to H&E staining to analyze for osteolytic legion formation. Representative H&E-stained images are shown on the left (scale bar, 100 μm), and quantification of bone cavity area is shown on the *right*. Data are shown as mean ± SD (n = 6). *p* values were calculated using one-way ANOVAwith *post hoc* Tukey’s test for multiple comparisons. ∗∗*p* < 0.01 *versus* RANKL + B32B3. E, Sections of calvarial bone from (D) were subjected to TRAP staining and histological analysis. Representative images of TRAP-staining on the *left* (scale bar, 100 μm), and quantification of TRAP-positive cells are shown on the *right*. Arrowheads point to TRAP-positive mature osteoclasts. Data are shown as mean ± SD (n = 6). *p* values were calculated using one-way ANOVAwith *post hoc* Tukey’s test for multiple comparisons. ∗∗*p* < 0.01 *versus* RANKL + B32B3. *F*, total RNAs were isolated from calvarial bones, and the relative mRNA levels of IRF8, MAFB, TMEM119 genes were quantified by RT-qPCR using primers listed in Table S1. All transcription levels were normalized to those of GAPDH. Data are shown as mean ± SD (n = 6). *p* values were calculated using two-way ANOVAwith *post hoc* Tukey’s test for multiple comparisons. ∗∗∗*p* < 0.001 *versus* RANKL + B32B3.

## Discussion

VprBP was recently identified as a kinase that stimulates RANKL-induced osteoclast differentiation by catalyzing H2AT120p and inactivating anti-osteoclastogenic genes, thus playing a critical role in the pathogenesis of bone diseases through excessive bone resorption (17). The dependence of VprBP function on its stable occupancy at target genes also supports the notion that H2AT120p-mediated gene silencing constitutes a crucial epigenetic process for efficient osteoclast differentiation. These observations were supportive of the idea that pharmacological inhibition of VprBP kinase activity may disrupt this transcriptional repression program and provide effective strategies to control osteoclast differentiation and bone resorption. In accordance with this idea, our previous investigation successfully used B32B3 as an inhibitory tool for unraveling the mechanistic basis for how VprBP-mediated H2AT120p regulates gene transcription during RANKL-induced osteoclast differentiation (17). However, B32B3 exibits potent efficacy against VprBP only at high concentrations, and its use in the treatment of resorption-related bone diseases requires the development of more potent B32B3 mimicking inhibitors. In this study, we discovered the highly potent second-generation VprBP inhibitor that negatively affects osteoclast differentiation in much more efficient ways than the first-generation inhibitor B32B3. Among the 211 B32B3 derivatives that we prepared, our systematic characterization identified B1486 that exhibits the inhibitory activity in OCP-induced cells at concentrations far below those needed for B32B3. The following are the main features of B1486: (a) it has about 50 time greater potency than B32B3 in supressing RANKL-induced osteoclast differentiation; (b) it shows a strong and continuous inhibition of VprBP-mediated H2AT120p as measured in kinase assays; (c) it is much more effective than B32B3 in reactivating anti-osteoclastogenic genes which are epigenetically silenced by VprBP-mediated H2AT120p; and (d) it eliminates H2AT120p without affecting VprBP occupancy at target loci in OCP-induced cells. The observation that knockdown of VprBP generates similar outcomes through its inhibitory effects on H2AT120p led us to suggest that B1486 exerts its anti-osteoclastogeinc action through mainly targeting VprBP kinase activity toward H2AT120.

The reason for the increased efficacy of B1486 is not clear to us at this time. B1486 and B32B3 are similar ATP-competing compounds, but a major difference is additional alkyl substituent on the upper heterocycle of B1486. This might improve hydrophobic contact and stabilize a favorable binding orientation within the target poket, resulting in a stronger inhibitory effect of B1486 compared with B32B3. Also note, B6756 and B3703 were able to inhibit VprBP kinase activity in OCP-induced cells, but only at doses 8 to 10 times higher than that of B1486. Further studies are required to understand the molecular basis for differential VprBP impairing properties of those two compounds, but it might reflect the existance of VprBP in a conformation that is less compatible with those inhibitor binding in OCP-induced cells. In any case, understanding why B1486 has higher inhibitory effects than B6756 and B3703 could shed light on how different inhibitors develop mechanisms through which they can be more potent against VprBP and exhibit better efficacy in attenuating H2AT120p in OCP-induced cells.

In a complementary approach, we used zebrafish as a vertebrate model to evaluate B1486 at concentrations where it effectively blocks RANKL-induced osteoclast differentiation *in vitro*. Our study showed that B1486 was superior to B32B3 in suppressing osteoclast formation and bone resorption in response to PDS treatment. In contrast, the same concentrations of B6756 and B3703 only moderately rescued a defective bone development in PDS-treated zebrafish (not shown). In testing B1486 in a mouse model, we also found that B1486 at a dose of 0.1 mg kg^–1^ significantly inhibits bone resorption, thereby interfering with bone destruction and osteoporosis. We believe that this is due to the capability of B1486 to block H2AT120p-dependent function of VprBP, as the dose used in this study resulted in nearly complete loss of H2AT120p at target genes. Moreover, the observation that B1486 is well tolerated by mice with no body weight loss suggests that VprBP-targeted action of B1486 may provide more efficacious and potent anti-osteoclastogenic therapy. In this regard, it should be emphasized that our study provides the basis for the use and optimization of VprBP inhibitors as epigenetic agents that modulate osteoclastogenic gene silencing. In some biological evaluations, kinase-targeted inhibitors are found to be nonspecific inhibitors for the management of gene misregulation. However, this is not the case for B1486, because B1486 has a particular preference for VprBP, and this preference can be further tuned by optimizing analogs with greater activity even when the mechanism of target gene suppression through H2AT120p is not completely understood. By extensively characterizing B1486 and directly addressing its limitations while preserving the suppression of VprBP kinase activity, our future study will provide the basis for the development of more potent VprBP inhibitors to prevent and treat bone loss as anti-resorptives. Given that VprBP plays a role in tumorigenesis, especially in the development of colon cancer and melanoma, our findings also have important clinical implications by raising the prospect that B1486 may also be optimized for the potential use as cancer therapeutics.

## Experimental procedures

### Antibodies

Antibodies used in this study are as follows: anti-H2A antibody form Abcam; anti-H2AT120p antibody from Active Motif; and anti-VprBP antibody from Proteintech. All antibodies used in this study were validated according to the manufature’s specification and are registered with a Research Resorce Identifiers (RRIDs) to ensure reproducibility.

### Osteoclast differentiation and Western blotting

Osteoclast precursor (OCP) cells were prepared as recently described (17, 20, 21, 22). To generate osteoclasts, OCP cells were cultured in MEM containing 10% fetal bovine serum (FBS) supplemented with 30 ng/ml macrophage colony-stimulating factor (M-CSF) and 50 ng/ml receptor activator of nuclear factor kappaB ligand (RANKL). On days 0, 1, 3, and 5, the cells were fixed with formaldehyde and stained for tartrate-resistant acid phosphatase (TRAP) using an acid phosphatase leukocyte kit (Sigma). TRAP-positive multinucleated cells containing three or more nuclei were counted as osteoclasts under a light microscope. Whole cell lysates were prepared from OCP-induced cells using M-PER Mammalian Protein Extraction Reagent (Thermo Fisher Scientific) according to the manufacturer’s instructions. Chromatin fraction was extracted from OCP-induced cells following the protocol described in our previous studies (11, 12, 15, 17, 20, 21, 22, 23). Western blot analysis was performed with prepared samples as described previously (11, 12, 15, 17, 20, 21, 22, 23). Briefly, the protein samples were separated using 15% SDS-PAGE (Bio-Rad) and transferred to a 0.45 μM nitrocellulose membrane (GE Healthcare). After blocking with 5% non-fat dry milk (Rockland), the membrane was incubated at 4°C with a primary antibody and washed with 0.05% Tween-20 in TBS (TBS-T). The membrane was washed with TBS-T, incubated at room temperature with a secondary antibody for 1 h, and protein bands were detected using an ECL reagent (Thermo Fisher Scientific) and visualized using a cheminuminescence imaging system. The band intensities of protein were quantified using Image J software (v1.53k) after normalization to Actin or H2A signals. The corresponding optical densities are shown below.

### Pooled and individual compound screening

A pooled screening approach was employed to identify highly potent VprBP inhibitors from a library of 211 B32B3 mimicking small-molecule compounds. The compounds were grouped into non-overlapping pools of five, with each compound applied at a final concentration of 100 nM, resulting in a total concentration of 500 nM per pool. OCP-induced cells were treated with the compound pools under standard differentiation conditions for 5 days. DMSO-treated cells were used as a negative control, while 100 nM B32B3-treated cells were used as a positive control to evaluate specific inhibitory effects of 211 compounds on osteoclastogenesis. In our evaluation, osteoclast differentiation was assessed using a TRAP activity assay, and TRAP-positive multinucleated cells containing three or more nuclei were counted as osteoclasts under a light microscope. Following the primary screening, three pools (Mix 6, Mix 11, and Mix 13) exhibiting significant inhibition of osteoclastogenesis relative to DMSO controls were selected for further evaluation to identify the active compounds within each pool. The inhibitory efficacy of individual compounds from the selected pools was assessed relative to a positive control group treated with 100 nM B32B3 under identical differentiation conditions for 5 days. From these analyses, B1486, B6756, and B3703 compounds were identified as being highly potent and further tested individually at 1, 10, and 100 nM for 5 days in dose–response experiments. All experiments were performed under identical conditions, including the same cell batch, vehicle, and treatment schedule, and were repeated in at least three independent experiments.

### CATS molecular descriptor

Descriptor calculation was performed with a proprietary Java-based software tool. The software is provided at URL (http://modlab-cadd.ethz.ch/).

### Cell viability assay

OCP-induced cells were cultured in 96-well plates at a density of 5 × 10^3^ in the absence or presence of VprBP inhibitors for 5 days. Cell viability was assessed by using the cell proliferation reagent WST-1 (Roche Diagnostics) as detailed previously (12, 15).

### *In vitro* kinase assay

Nucleosomes were reconstituted using the 601-nucleosome positioning sequence and recombinant histones as recently described (24, 25, 26, 27). For *in vitro* kinase assays, recombinant VprBP was incubated with reconstituted nucleosomes (2 μg) in kinase buffer (50 mM Tris-HCl [pH 7.5], 20 mM EGTA, 10 mM MgCl_2_, 1 mM DTT, and 1 mM b-glycerophosphate) containing 4 mM ATP as recently described (11, 15, 16). Following incubation for 30 min at 30 °C, H2A proteins from each reaction were separated by SDS-PAGE, and phosphorylated H2A proteins were analyzed by Western blotting. All assays were conducted in triplicates.

### RT-qPCR

Total RNA was isolated from OCP-induced cells by using the RNeasy Mini kit (Qiagen Inc) and converted to first-strand cDNA using the SuperScript III First-Strand System Kit (Thermo Fisher Scientific). Real-time RT-qPCR was performed with SYBR Green Real-time PCR Master Mixes (Thermo Fisher Scientific) according to the manufacturer’s instructions. The primers used for RT-qPCR are listed in Table S1. All mRNA values were normalized to GAPDH mRNA levels, and all reactions were run in triplicate.

### ChIP-qPCR

ChIP assays with OCP-induced cells were performed using the ChIP Assay Kit (Millipore) as recently described (15, 28). After reversing protein–DNA crosslinks, immunoprecipitated DNA was purified and analyzed by qPCR using the primers that amplify the promoter (P), TSS, and coding region (C) of VprBP target genes. The primers used for qPCR are listed in Table S2. Specificity of amplification was determined by melting curve analysis, and all samples were run in triplicate.

### VprBP inhibitor treatment in zebrafish and calcein labeling

Zebrafish (*Danio rerio*, AB strain) were raised in a recirculating water system according to previously established protocols (17, 29, 30). All zebrafish were maintained under a 14 h/10 h light-dark cycle with pH of 7.0 ± 1.0 and 28 ± 0.5°C. To obtain embryos, adult males and females were placed in a specialized breeding tank. The following morning, embryos were collected and cultured in petri dishes containing E3 water (5.0 mM NaCl, 0.17 mM KCl, 0.33 mM CaCl_2_, and 0.33 mM MgSO_4_). At 6 dpf, zebrafish larvae were treated with 25 μM PDS in the absence or presence of DMSO, B32B3, or B1486 for 3 days. The concentrations of B32B3 and B1486 were set at 1/50 of the effective *in vitro* doses based on our results (Fig. 2). These concentrations were expected to be pharmacologically relevant without causing any toxic reactions, including abnormal morphology, impaired motility, or mortality. For calcein staining, a fluorescent dye staining method commonly used to acess osteogenic levels (30, 31), zebrafish larvae were collected at 9 dpf and immersed in a 1% working solution of calcein (Sigma) for 10 min. Subsequently, the embryos were washed three times with E3 water (3 min per wash) to remove any unbound dye. The calcein-stained vertebral column within the spinal region of zebrafish larvae was analyzed using fluorescence microscopy at 10× magnification. Imaging was performed with a long-pass green filter set for excitation at 480 ± 40 nm and emission at 510 nm, allowing for precise visualization of mineralized structures. All zebrafish studies were approved by the Institutional Animal Care and Use Committee and conducted in accordance with the guidelines set forth by the committee.

### VprBP inhibitor treatment in mice

Eight-week-old C57BL/6 mice were obtained from Charles River Laboratories and housed in specific pathogen-free conditions. Mice were acclimatized under a constant light/dark cycle at 22 ± 2 °C (12 h light/dark cycles) and served with a general laboratory diet and water *ad libitum*. For the experiment, mice were randomly divided into three groups (n = 6 per group) and injected over the calvaria every other day with RANKL alone, RANKL + B32B3, or RANKL + B1486. On the intervening day, mice are treated with RANKL alone, for a total duration of 14 days. The doses of B32B3 and B1486 were determined based on our *in vitro* results, ensuring pharmacologically relevant effects while minimizing toxicity (Fig. 2). No signs of toxic reactions, including weight loss, abnormal behavior, or mortality, were observed at these doses. 14 days after injections, mice were sacrificed, and calvaria was collected and fixed with 4% paraformaldehyde. Paraffin-embedded calvaria sections (6 μm) were stained for hematoxylin/eosin and TRAP for histological analysis. Images were acquired with an Aperio ScanScope Model T3 and were analyzed with ImageScope software (Aperio Technologies) as described previously (17, 32, 33). All animal studies were conducted according to protocols approved by the Institutional Animal Care and Use Committee.

### Micro-CT analysis

Calvarial bones were harvested aseptically from euthanized mice and fixed in 4% paraformaldehyde as previously described (17, 32, 33). Micro-CT imaging was conducted using a SkyScan 1172 scanner (Bruker) at 67 kV, 147 μA, and 705 ms exposure time. Quantification of bone volume (mm^3^) normalized to tissue volume and assessment of bone mineral density within the defined regions were performed using CT-analyzer-based 2D/3D analysis platforms (Amira and Scanco). The regions of interest encompassed the coronal and sagittal suture areas of the calvaria. Three-dimensional reconstructions were generated by importing segmented datasets into Vgstudio Max 3.3.2.

### Statistical analysis

All quantitative data are presented as mean ± standard deviation (SD). Statistical analyses of datasets were performed with Student’s two-tailed *t* test, one-way ANOVA, or two-way ANOVA followed by Tukey’s *post hoc* test using GraphPad Prism 9 (GraphPad Software Inc). A *p* value < 0.05 was considered statistically significant.

## Data availability

All relevant data are available from the authors upon request.

## Supporting information

This article contains supporting information.

## Conflict of interest

The authors declare that they have no conflicts of interest with the contents of this article.
