# Supplementary material for: B1486 is a highly potent VprBP inhibitor capable of blocking osteoclast differentiation and bone resorption
Source: J Biol Chem. 2026 Sep 8;302(10):113524. doi: 10.1016/j.jbc.2026.113524 (PMC13671033; doi:10.1016/j.jbc.2026.113524)
Supplement: Supplementary file 1 [file mmc1.pdf]

SUPPLEMENTARY INFORMATION

B1486 is a highly potent VprBP inhibitor capable of blocking osteoclast differentiation and bone resorption

Sungmin Kim[1†], Yonghwan Shin[1†], Nikhil B. Ghate[1] and Woojin An[1*]

[1]Department of Cancer Biology, Norris Comprehensive Cancer Center, University of Southern California, Los Angeles, CA 90033, USA

[†]These authors contributed equally to this work

*Correspondance: woojinan@usc.edu

SUPPLEMENTARY FIGURES

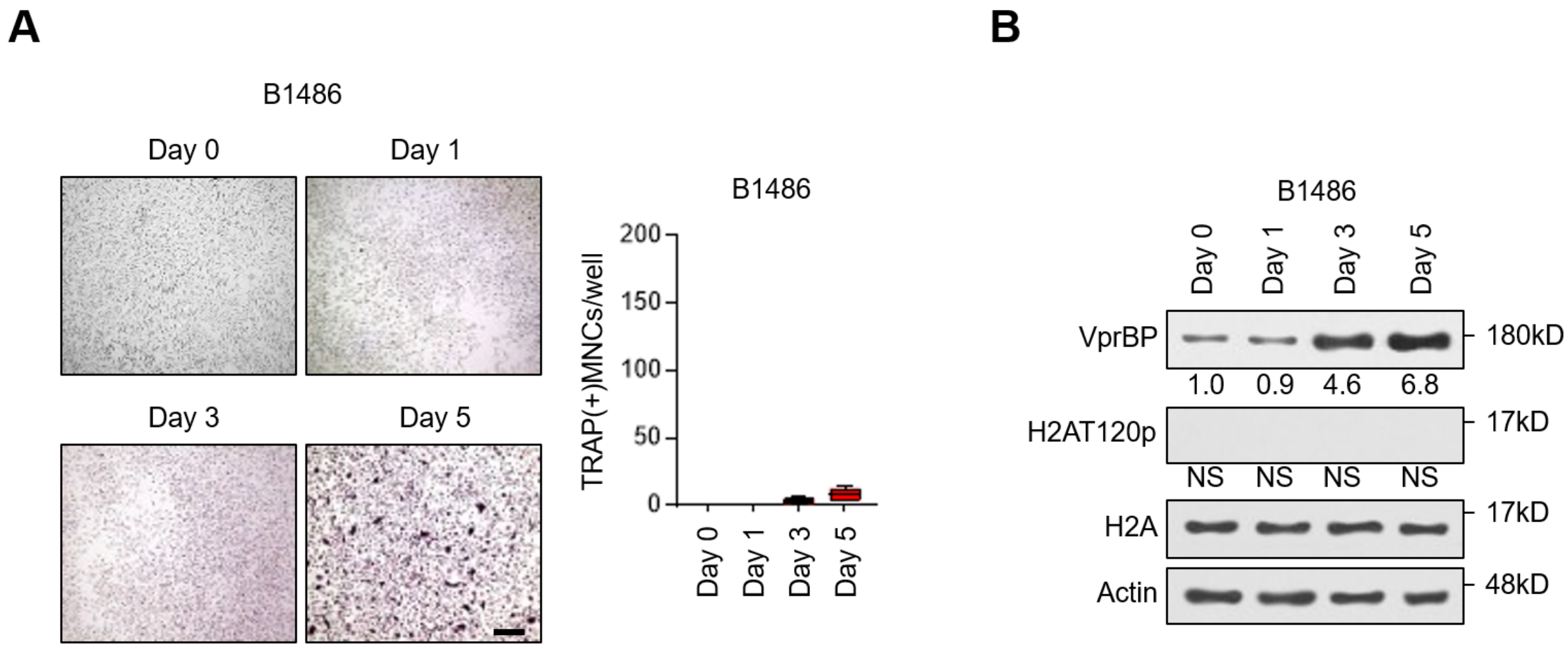

Figure S1. RANKL-induced osteoclast differentiation impaired by B1486

A, OCP-induced cells were cultured in the presence of VprBP inhibitor B1486 for 0, 1, 3, and 5 days fixed with formaldehyde, stained for tartrate-resistant acid phosphatase (TRAP) (left panel; scale bar, 100 μm), and counted under a microscope (right panel). Shown are the representative results of three independent biological experiments, each consisting of at least n = 5 technical replicates.

B, Whole cell lysate and chromatin fractions were prepared from OCP-induced cells and analyzed by Western blotting with VprBP, H2AT120p, and H2A antibodies. The band intensities of VprBP and H2AT120p were quantified using Image J software (v1.53k) after normalization to Actin and H2A signals. NS indicates no signal. Data are representative of three independent biological experiments.

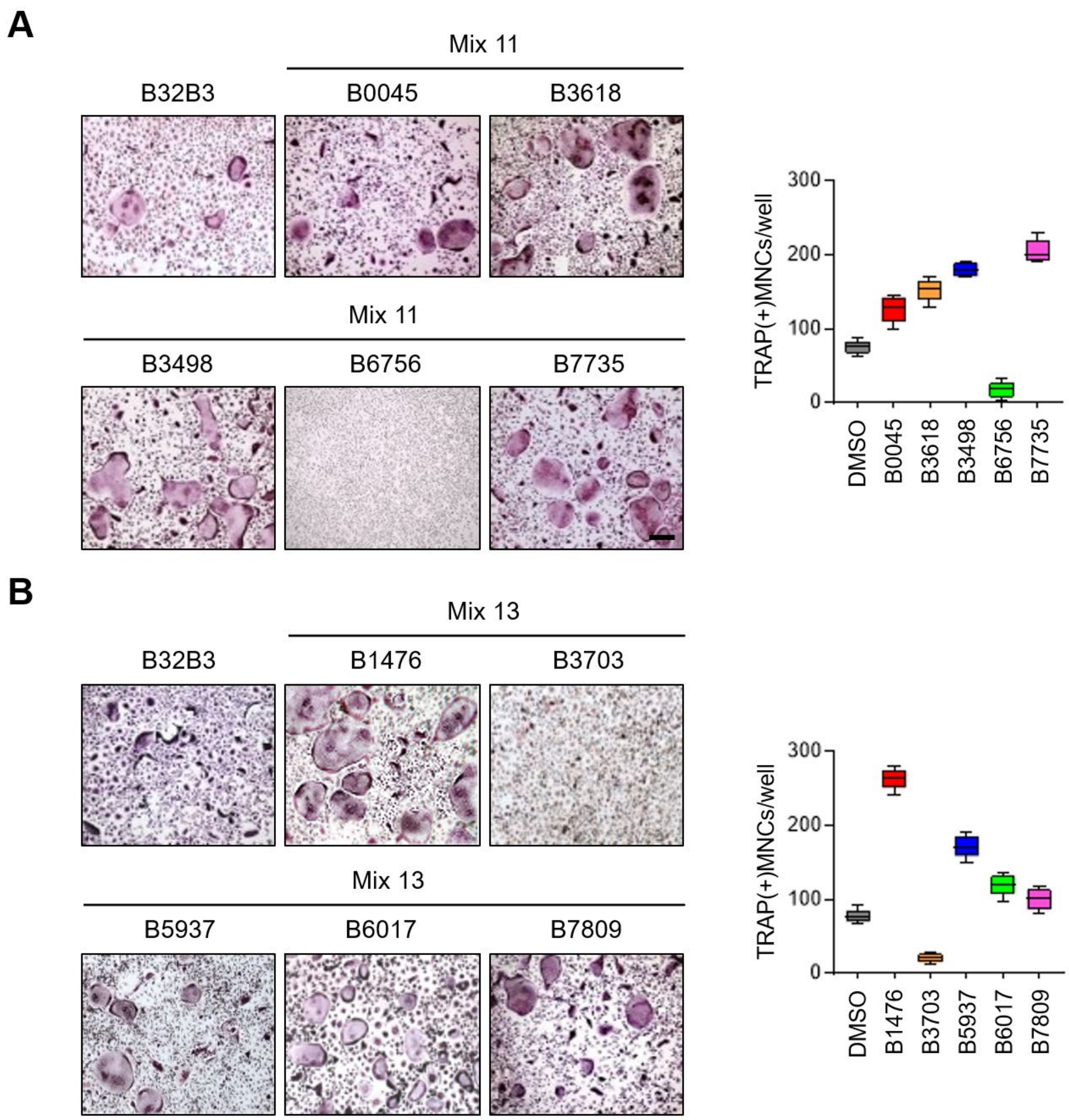

Figure S2. Effects of individual compounds of mix 11 and 13 on osteoclastogenesis. OCP-induced cells were grown in the presence of 100 nM of each indicated mix 11 (*A*) and mix 13 (*B*) compound for 5 days, and subjected to TRAP staining for microscopic evaluation (left panel; scale bar, 100 μm) and quantification (right panel). TRAP-positive cells containing three or more nuclei were counted as osteoclasts (right panel). Data represent the mean ± SD of three independent biological experiments, each consisting of at least n = 5 technical replicates. All experiments were performed under identical conditions, including the same cell batch, vehicle, and treatment schedule.

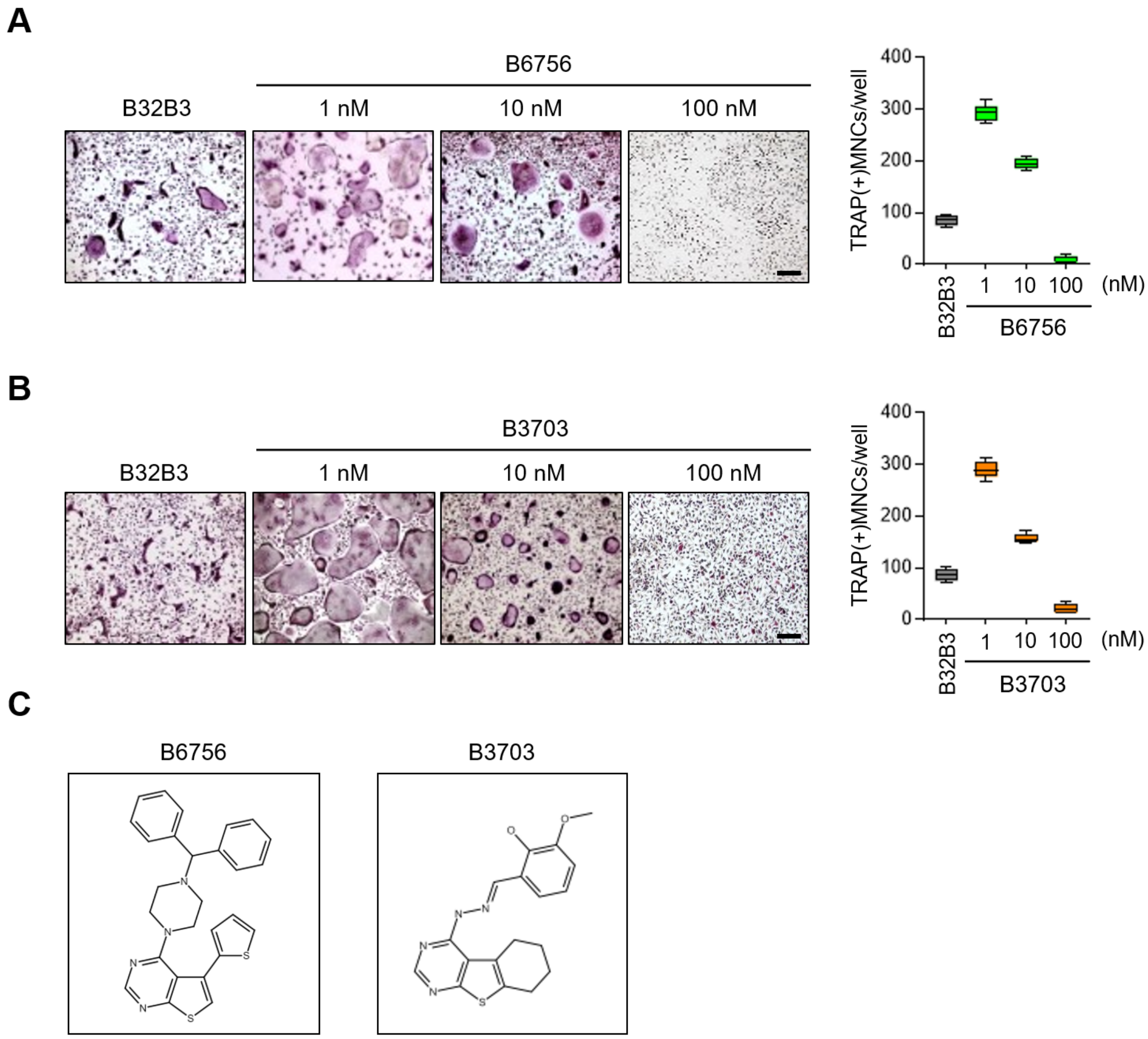

Figure S3. Effects of B6756 and B3703 on osteoclast differentiation.

OCP-induced cells were cultured with 100 nM B32B3 or the indicated concentrations of B6756 (*A*) or B3703 (*B*) for 5 days, and TRAP-stained for microscopic analysis (left panel; scale bar, 100 μm). TRAP-positive cells containing three or more nuclei were counted as osteoclasts (right panel). Data are represented as mean ± SD of three independent biological experiments, each consisting of at least n = 5 technical replicates. All experiments were performed under identical conditions, including the same cell batch, vehicle, and treatment schedule.

C, Molecular structures of B6758 and B3703.

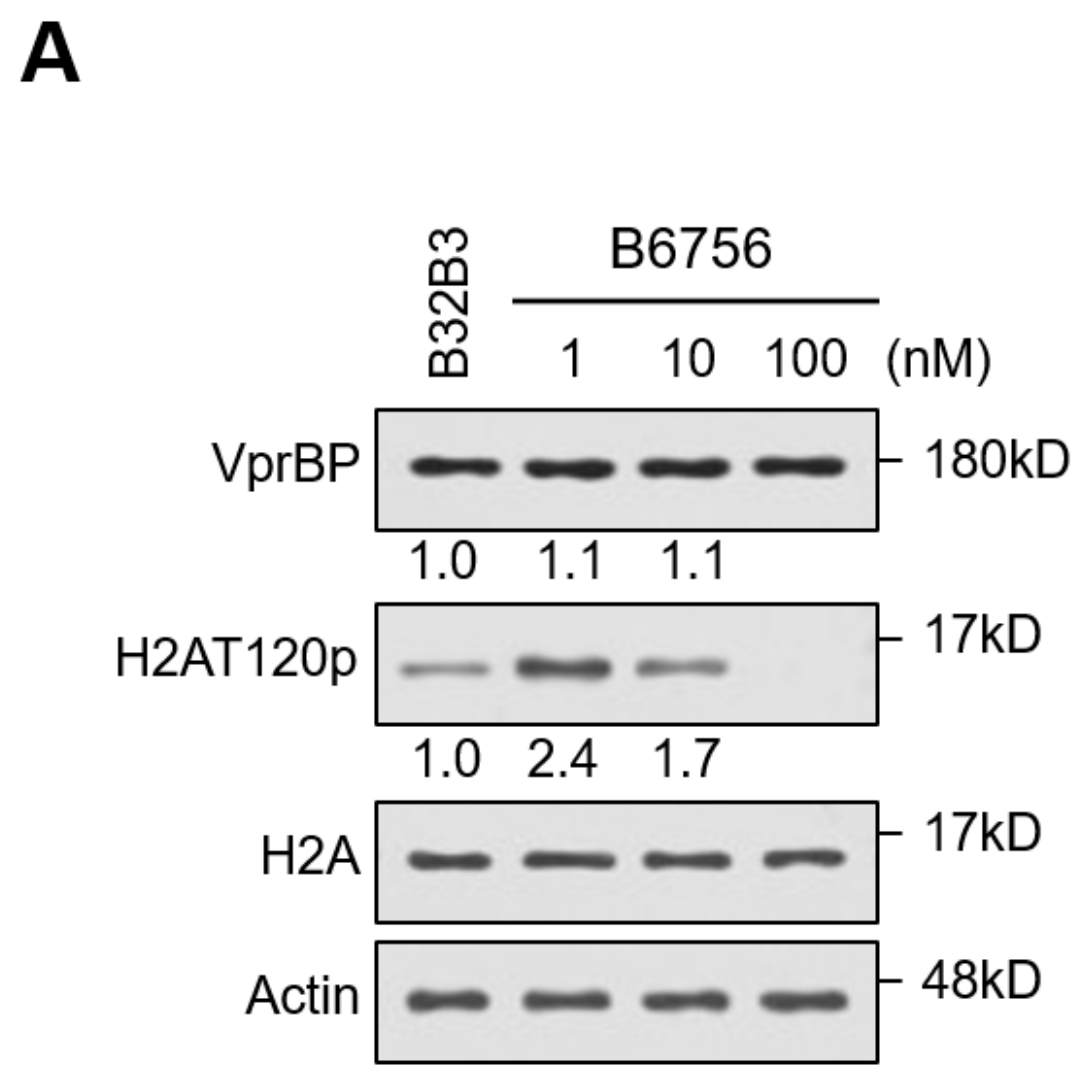

Figure S4. Effects of B6756 and B3703 on VprBP-mediated H2AT120p.

OCP-induced cells were treated with B6756 (*A*) or B3703 (*B*) as in Fig. S3, and whole cell lysate and chromatin fractions were extracted and analyzed by Western blotting with VprBP, H2A, and H2AT120p antibodies. The band intensities of VprBP and H2AT120p were quantified using Image J software (v1.53k) after normalization to Actin and H2A signals. NS indicates no signal. Data are representative of three independent biological experiments.

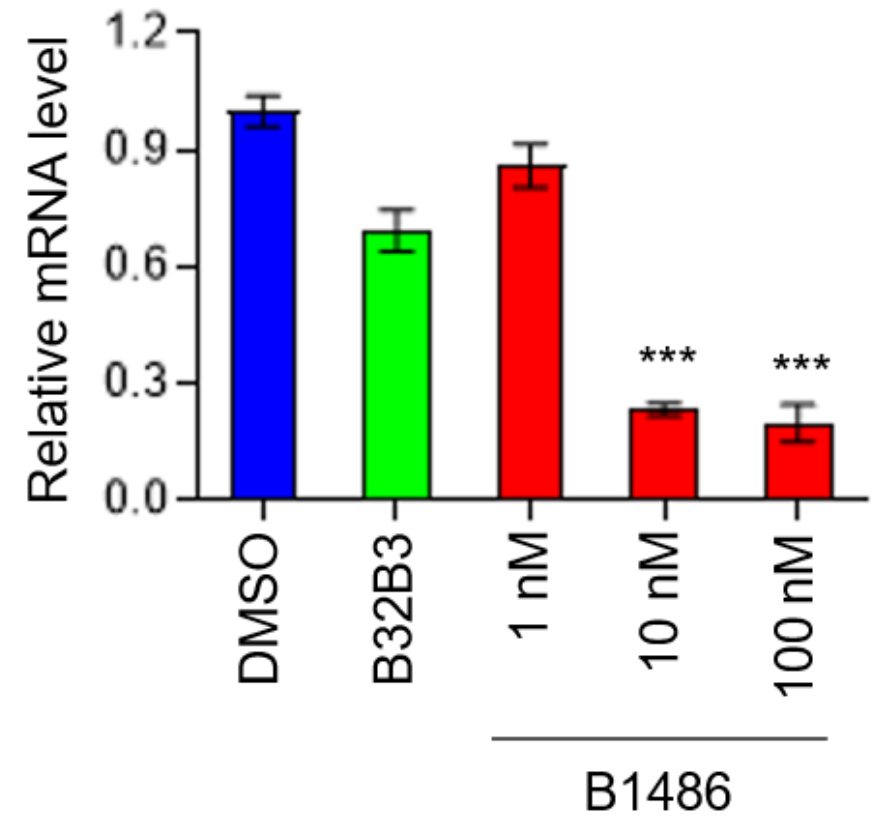

Figure S5. Effects of B32B3 and B1486 on osteoclastogenic gene expression in OCP-induced cells.

OCP-induced cells were cultured in the presence of B32B3 (100 nM) or B1486 (1, 10, or 100 nM) for 5 days. RNA samples were prepared, and NFATc1 expression was analyzed by RT-qPCR. All transcription levels were normalized to those of GAPDH. Data represents the mean ± SD of three independent biological experiments. *P* values were calculated using one-way ANOVA with post-hoc Tukey's test for multiple comparisons. ***$p < 0.001$ versus B32B3.

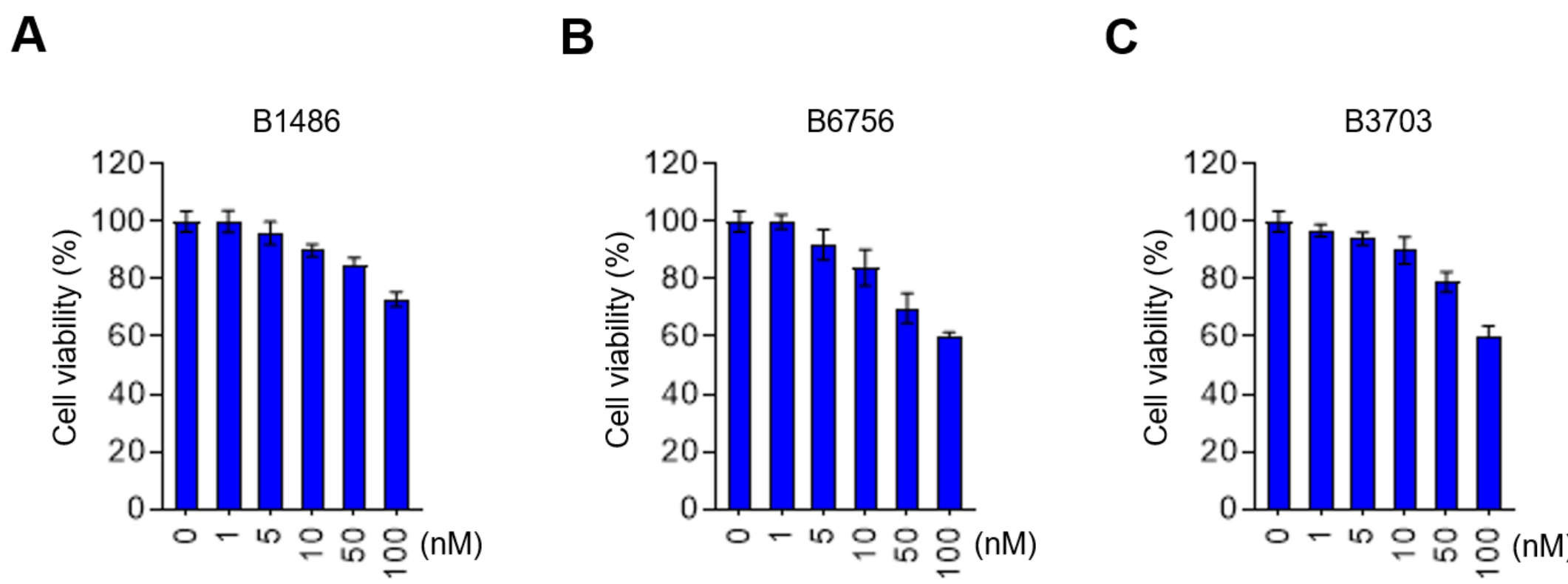

Figure S6. Effects of B1486, B6756, and B3703 on OCP cell viability.

OCP-induced cells were grown in the presence of the indicated concentrations of B1486 (*A*), B6756 (*B*), or B3703 (*C*), and their relative viability was assessed using the cell proliferation reagent WST-1. Data are represented as mean ± SD of three independent biological experiments.

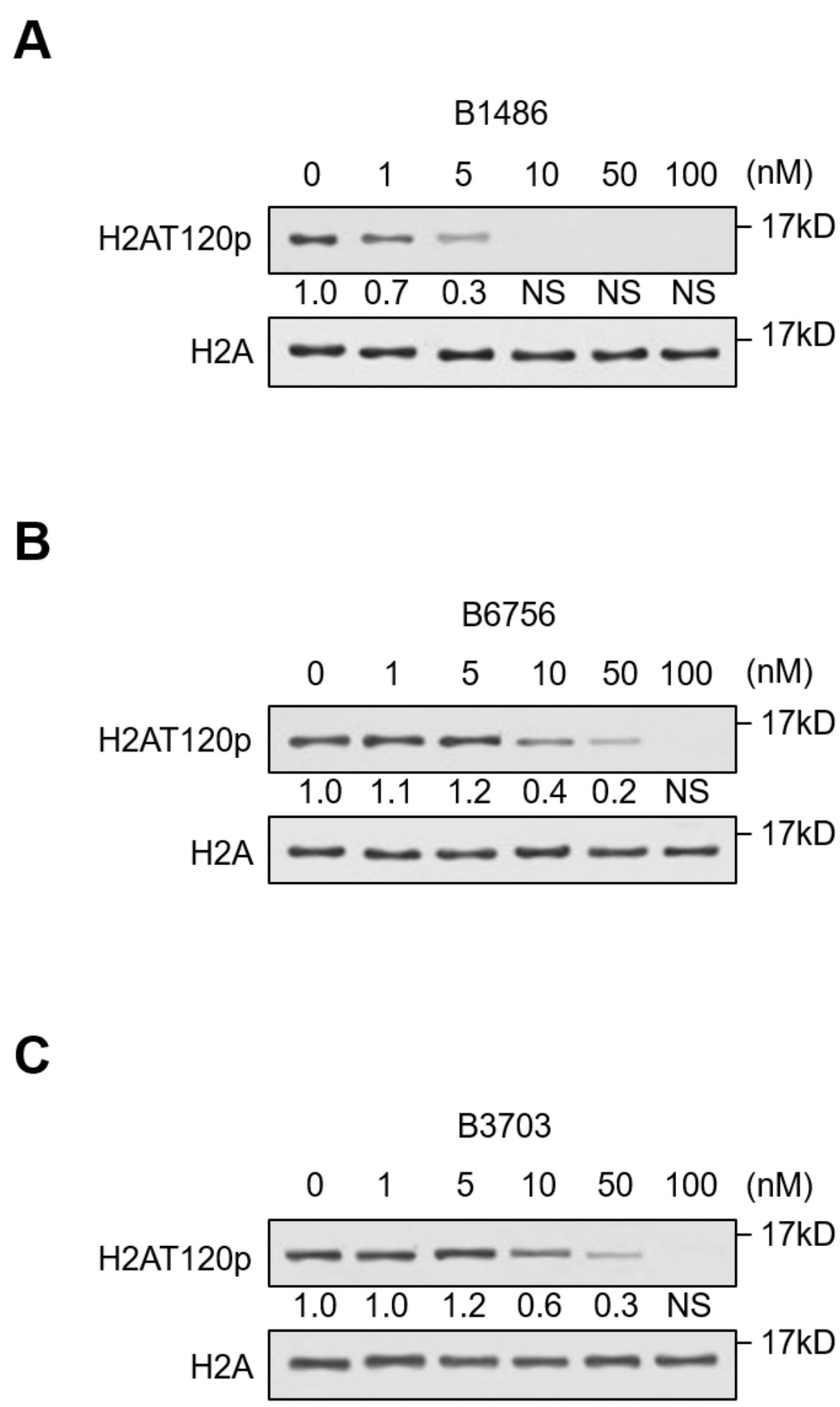

Figure S7. Effects of B1486, B6756, and B3703 on recombinant VprBP

Nucleosomes were reconstituted on the 601 nucleosome positioning sequence with recombinant histone octamers and subjected to in vitro kinase assays with recombinant VprBP in the presence of the indicated concentrations of B1486 (*A*), B6756 (*B*), and B3703 (*C*). The effects of the compounds were evaluated by Western blotting with H2A and H2AT120p antibodies. The band intensities of VprBP and H2AT120p were quantified using Image J software (v1.53k) after normalization to H2A signals. NS indicates no signal. Data are representative of three independent biological experiments.

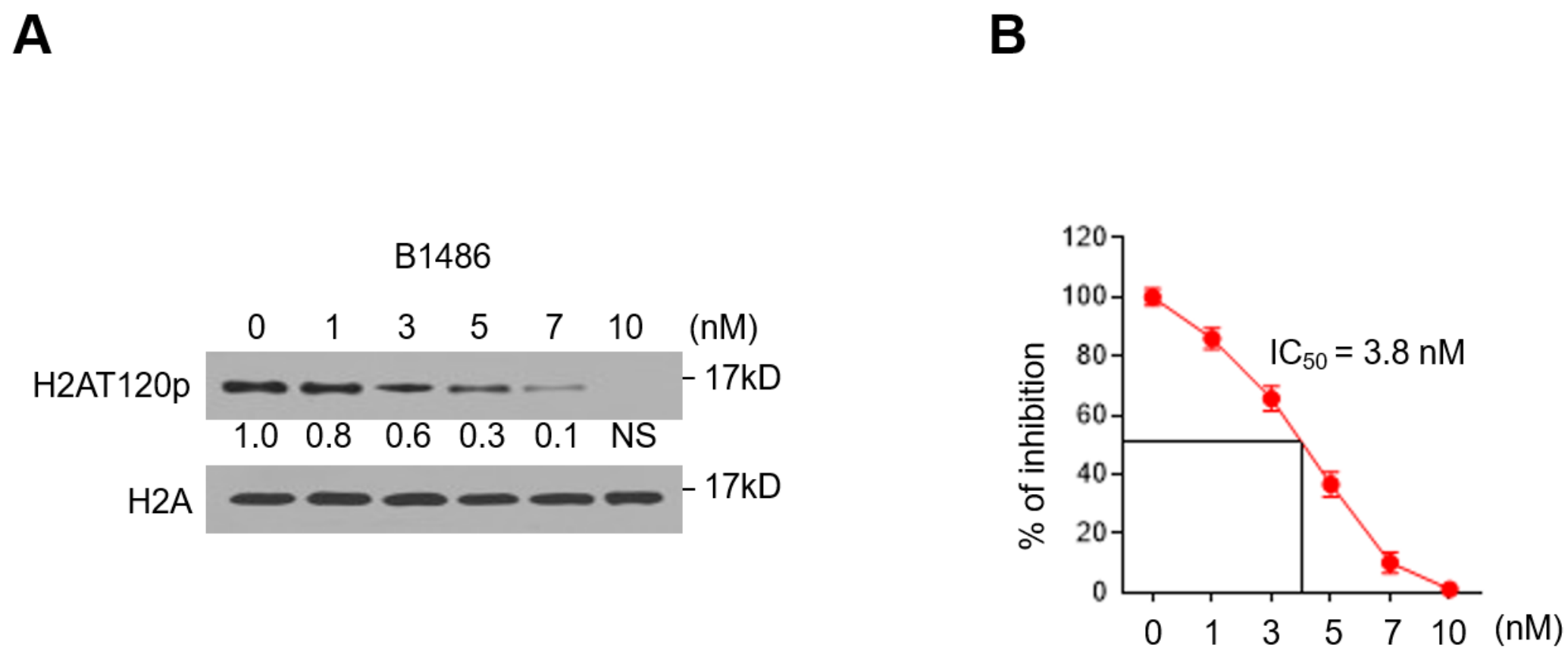

Figure S8. Determination of IC_{50} of B1486.

A, In vitro kinase assays were performed with VprBP and nucleosomes in the presence of increasing concentrations of B1486. The inhibitory effects of B1486 were evaluated by Western blotting with H2A and H2AT120p antibodies. The band intensities of VprBP and H2AT120p were quantified using Image J software (v1.53k) after normalization to H2A. NS indicates no signal. Data are representative of three independent biological experiments.

B, Dose-response curves were fitted using Graphpad Prism to determine IC50 value of B1486. Data are represented as mean ± SD of three independent biological experiments.

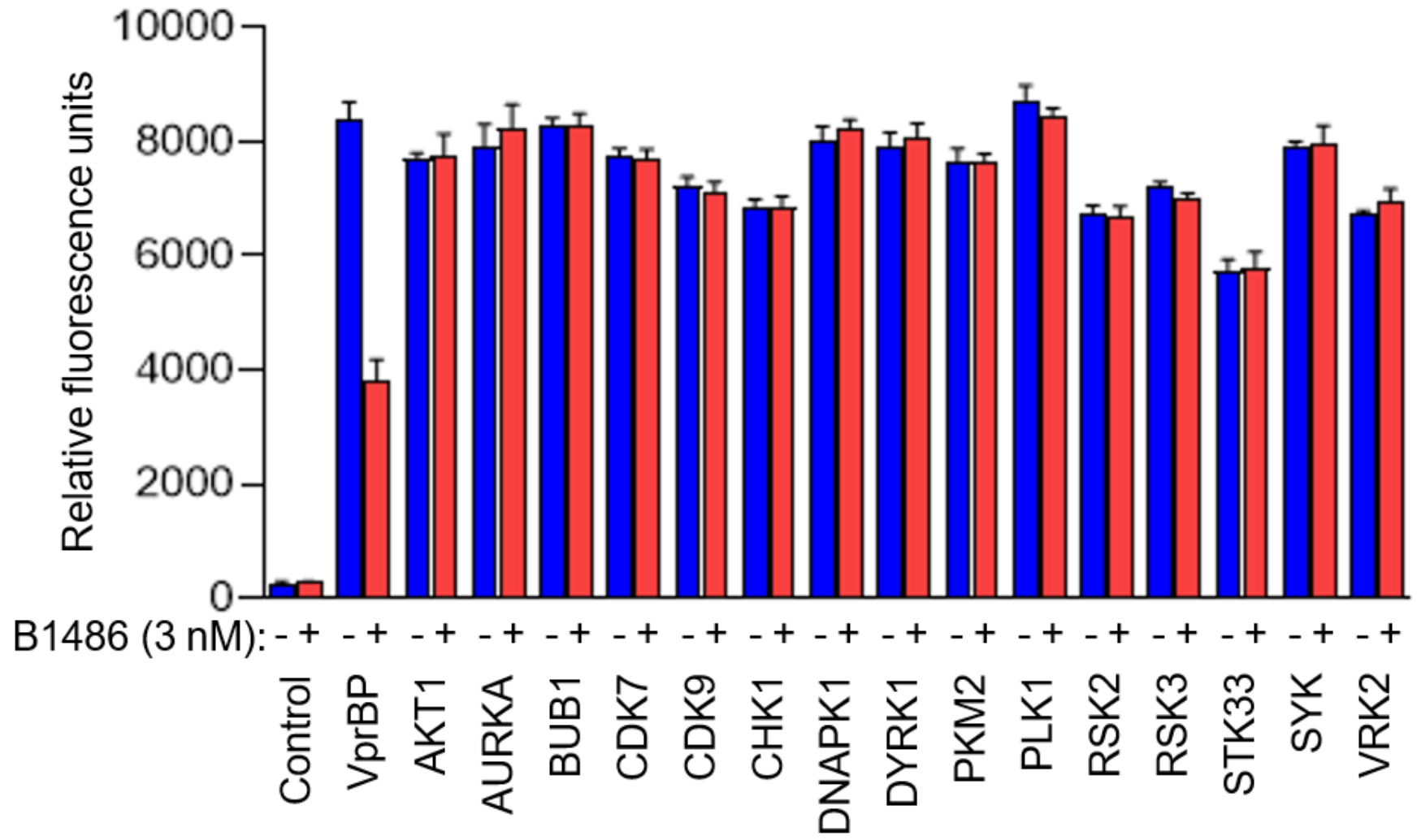

Figure S9. Analysis of B1486 selectivity.

A panel of 16 human protein kinases including VprBP were incubated with 1 μM ATP and their respective substrates at room temperature for 1 h, and kinase activity was quantified by measuring ADP production based on fluorescence intensity (Ex/Em = 540/590 nm). Data are represented as mean ± SD of three independent biological experiments.

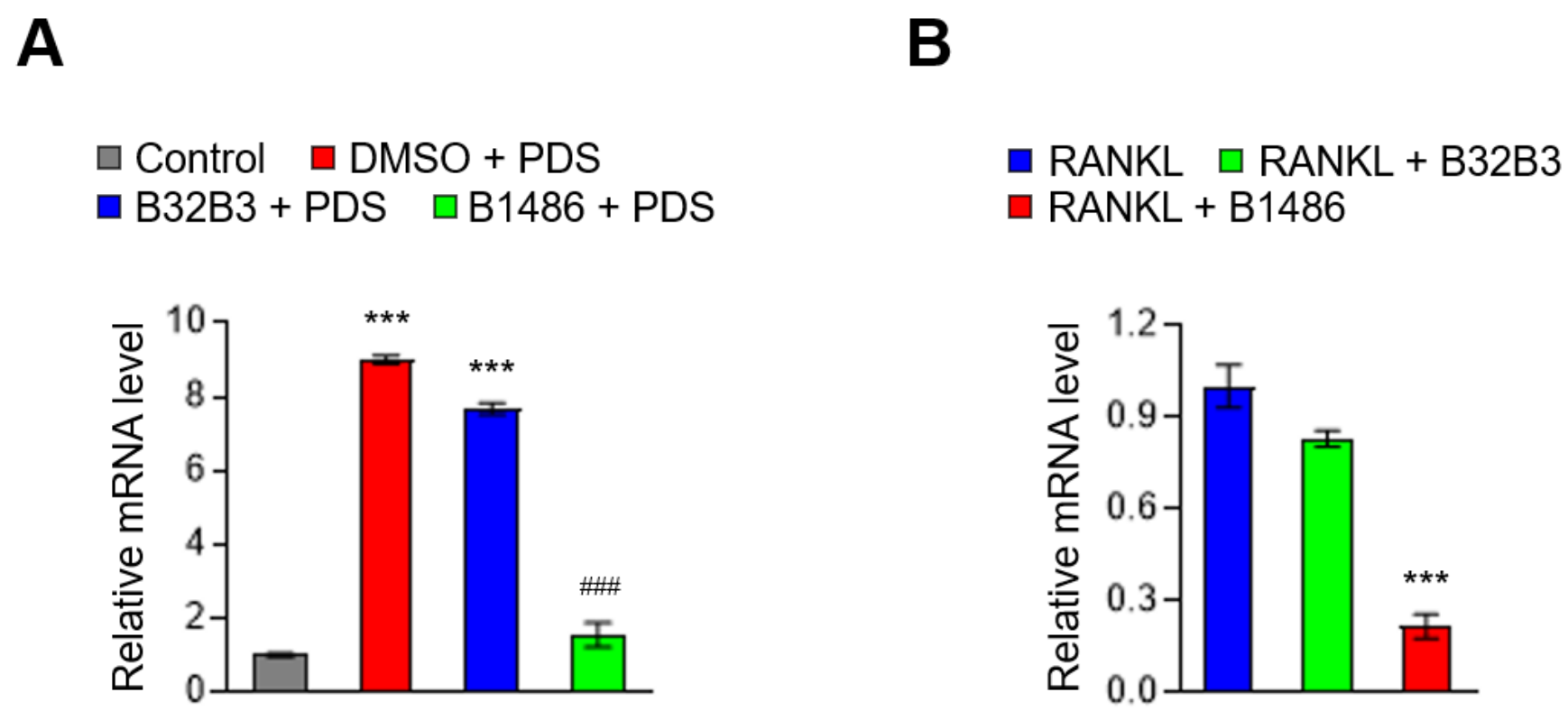

Figure S10. Effects of B32B3 and B1486 on osteoclastogenic gene expression in zebrafish and mouse model.

A, Zebrafish larvae were treated with 2 μM B32B3 or 40 nM B1486 in the presence or absence of 25 μM prednisolone from 6 days post fertilization (dpf) to 9 dpf. Total RNA was extracted, and NFATc1 expression was analyzed by RT-qPCR. Data represent the mean ± SD of independent triplicate experiments. ***p < 0.001 versus control; ###p < 0.001 versus B32B3 + PDS.

B, C57BL/6 mice (8 weeks old) were treated with RANKL alone (RANKL) or RANKL together with B32B3 (0.1 mg kg^{-1}; RANKL + B32B3) or B1486 (0.1 mg kg^{-1}; RANKL + B1486) every other day for 14 days. Total RNA was isolated from calvarial bones, and NFATc1 expression was analyzed by RT-qPCR. All transcription levels were normalized to those of GAPDH. Data are shown as mean ± SD (n = 6). *P* values were calculated using one-way ANOVA with post-hoc Tukey's test for multiple comparisons. ***p < 0.001 versus RANKL + B32B3.

SUPPLEMENTARY TABLES

Table S1. List of the primers used in RT-qPCR.

Primers	Forward (5´- 3´)	Reverse (5´- 3´)
ATOH8	TCAACGGAGATCAAAGCCCT	TGCCAGGGACAAGATGTAGT
CMBL	GCTGCCCAACACCAGGTATA	CAGCATCAACCTCCCGATTG
GAPDH	AACTTTGGCATTGTGGAAGG	GGATGCAGGGATGATGTTCT
GDPD2	GACTTCTCCTGGGCTGAACT	TTCTCAGCCTCCTTCCGATC
IRF8	GACCATGTTCCGTATCCCCT	TGGTTCAGCTTTGTCTCCCT
MAFB	GAGGACCGCTTCTCTGATGA	CTGGACGCGTTTATACCTGC
NEGR1	AACACACACCACGGACAATG	GCTGATGGGGAGATATGCCT
POLQ	TCCGGGTGTCCTTACTCAAC	CCTTCCGGGCACTTTTGAAA
RBMS3	ACAGATGGCCAAGCAACAAG	TATCACGTGTCCAAAGGGCT
SKA3	TTCAAAAGGCCGGTGTGAAG	GGGTCTCAGCTACACGTTCT
TMEM119	CAGCCTACTACCCATCCTCG	GTAGCAGCCAGAATGTCAGC
RPL23	ACGGCTGAACAGACTTCCTGCT	CGTTGTCGAATTACCACTGCTGG
NFATc1	GGTGCCTTTTGCGAGCAGTATC	CGTATGGACCAGAATGTGACGG
IRF8 (zebrafish)	TTCAGTGCAAGAATGGTGGC	TTGTCCACCATCTGACCTCC
MAFB (zebrafish)	AGCTTGTGACCATGTCCGTA	TCTTCTCGTTCTCCAGCAGG
TMEM119 (zebrafish)	CGTGGATGAGCAAGACAAGG	CCTTCCACGGACTCTTGAGT
NFATc1 (zebrafish)	TGTGAAAGCACTAGCGGGAG	TGGTAGAATGCGTGAGGTCG

Table S2. List of the primers used in ChIP-qPCR.

Primers	Forward (5´- 3´)	Reverse (5´- 3´)
IRF8 (P)	TCACCCTCAAAGACCCCATC	CACTAGAAGACACCAGCCCA
IRF8 (TSS)	TACAGGCTCT GATTGCAGGA	TCCTGCAATCAGAGCCTGTA
IRF8 (C)	GCTTCCATCTTCAAGGCCTG	GTTCAGAGCACAGCGTAACC
MAFB (P)	CCTCATCGCCCATCTCATCT	AACTGCTGGGGAAATCTCGA
MAFB (TSS)	AGCCCTAGAGCCAAAGAGTC	CGGGGCAGGGTAGAGAAG
MAFB (C)	CACCATCATCACCAAGCGTC	TCATCAGAGAAGCGGTCCTC
TMEM119 (P)	TGCAAGGCCAGGAGTAATGA	AGGGATGCATTTGGAGACCA
TMEM119 (TSS)	CCATAGCTCA ACATGGTCCC	GGGACCATGTTGAGCTATGG
TMEM119 (C)	TTCTTCCGGCAGTACGTGAT	CGAGGATGGGTAGTAGGCTG
